# Supplementary material for: MiR-525-5p inhibits diffuse large B cell lymphoma progression via the Myd88/NF-κB signaling pathway
Source: PeerJ. 2023 Nov 6;11:e16388. doi: 10.7717/peerj.16388 (PMC10634338; doi:10.7717/peerj.16388)
Supplement: Supplemental Information 3 [file peerj-11-16388-s003.docx]

**Figure 3D GAPDH**


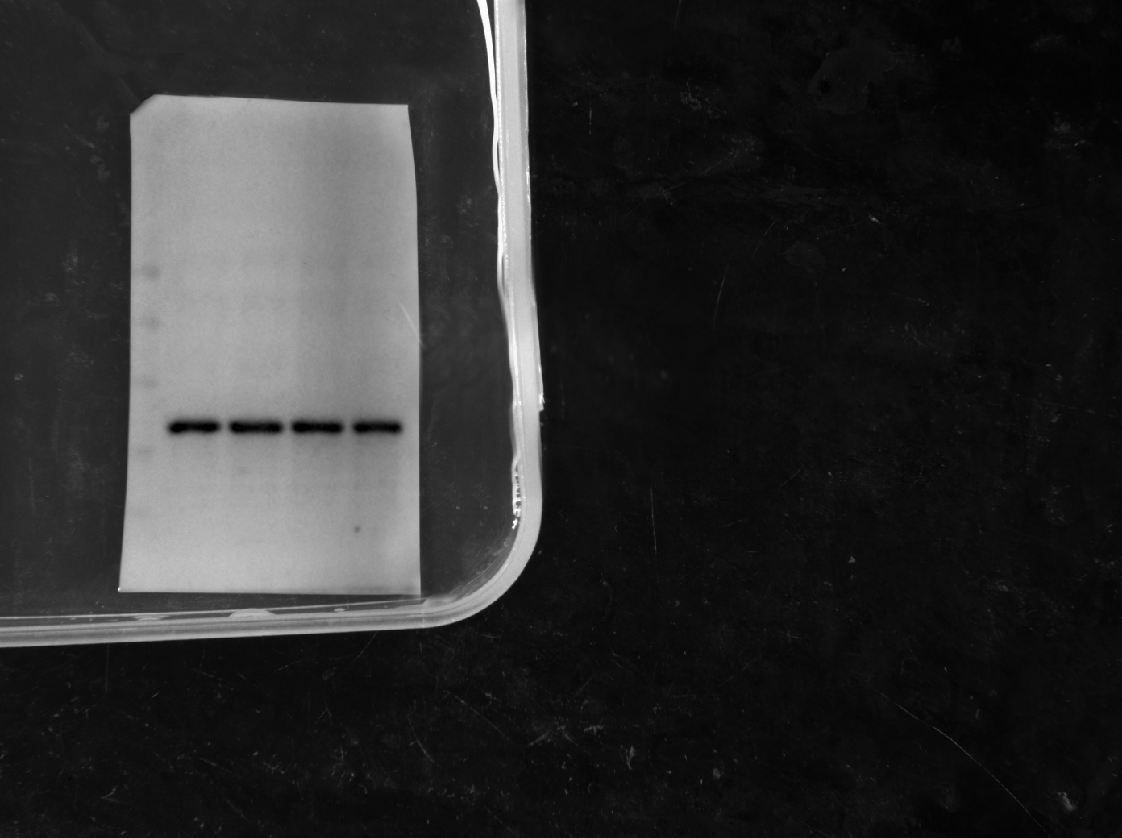


**Figure 3D Myd88**


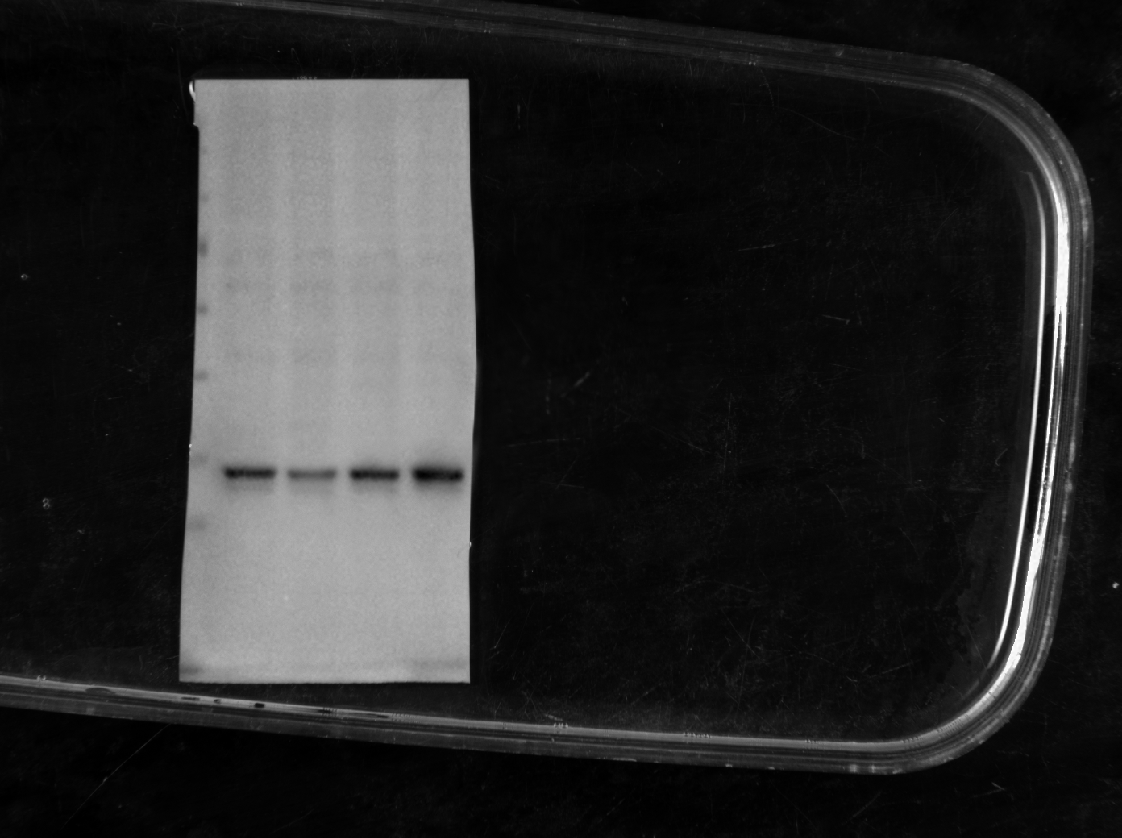


**Figure 3E GAPDH**

**
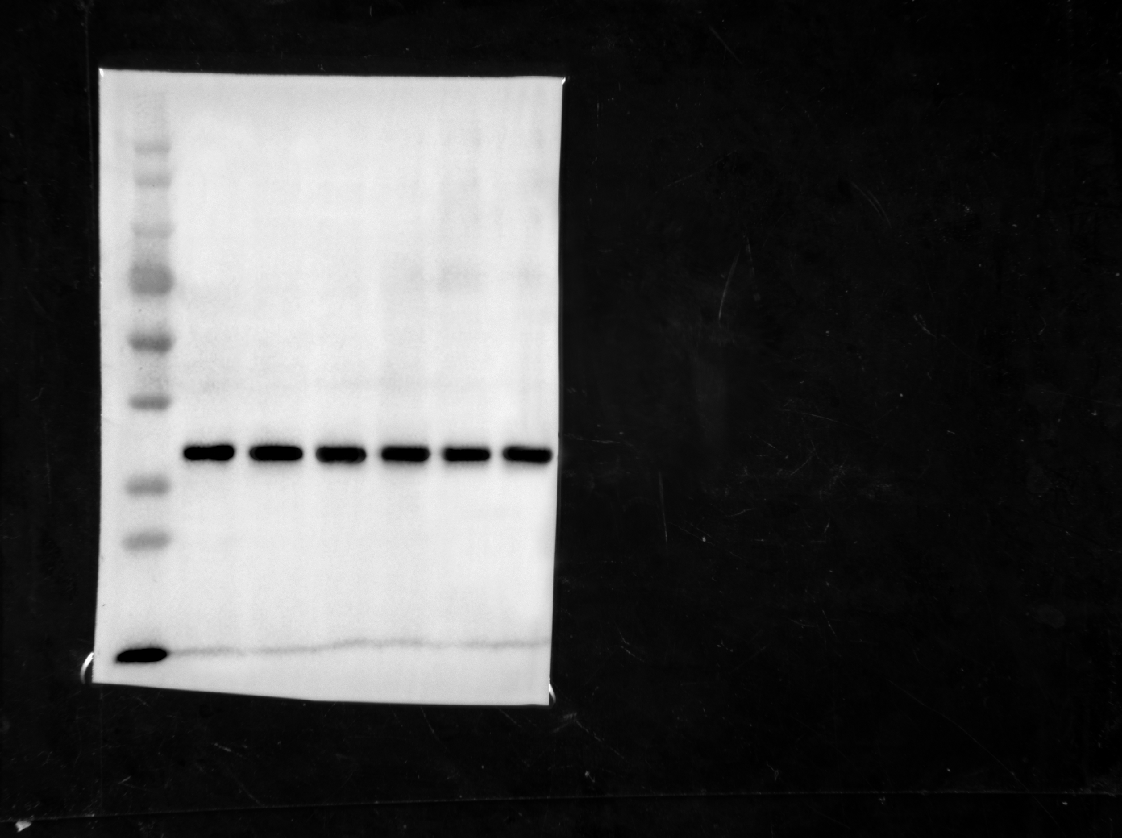
**

**Figure 3E Myd88**

**
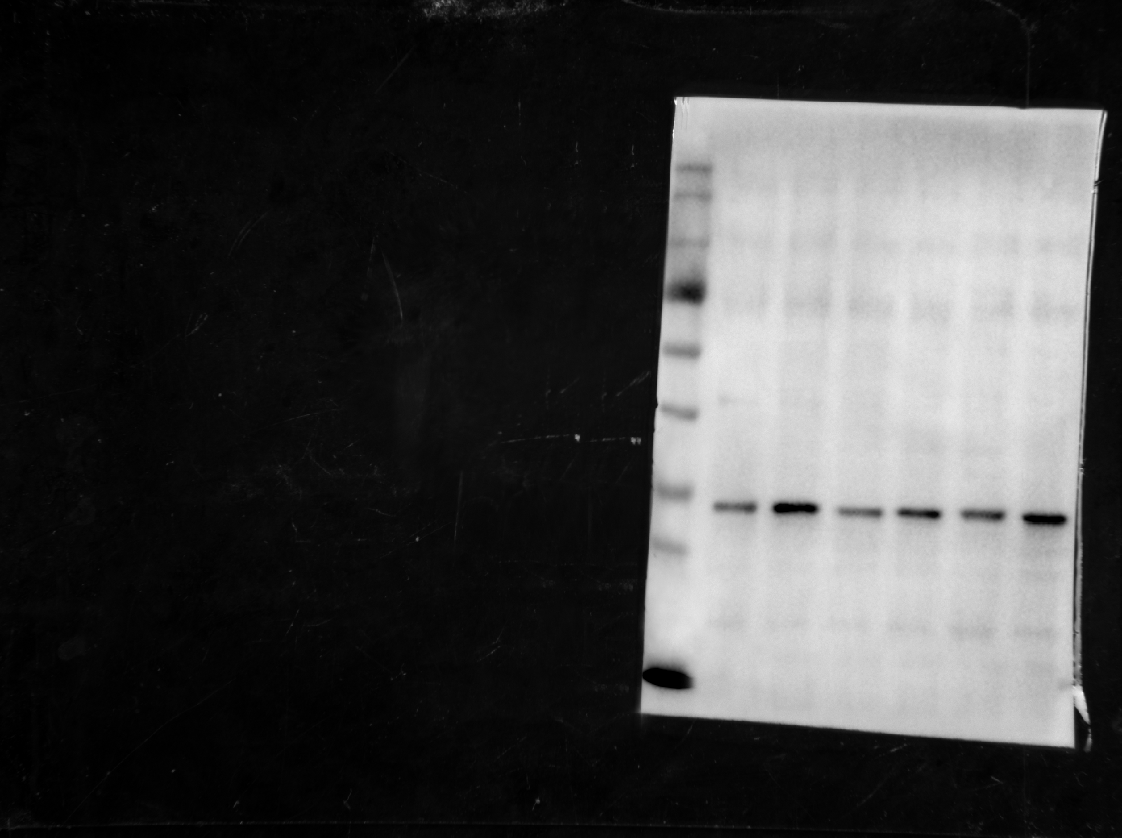
**

**Figure 4A GAPDH**

**
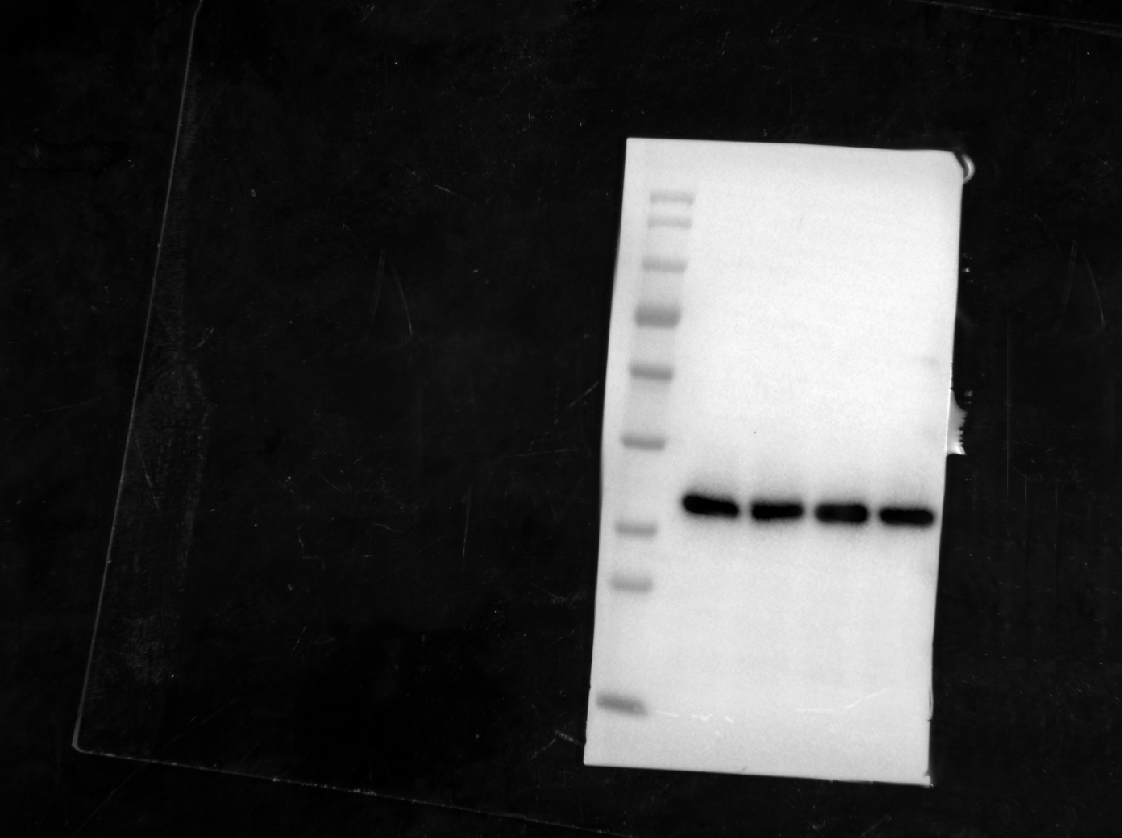
**

**Figure 4A Myd88**

**
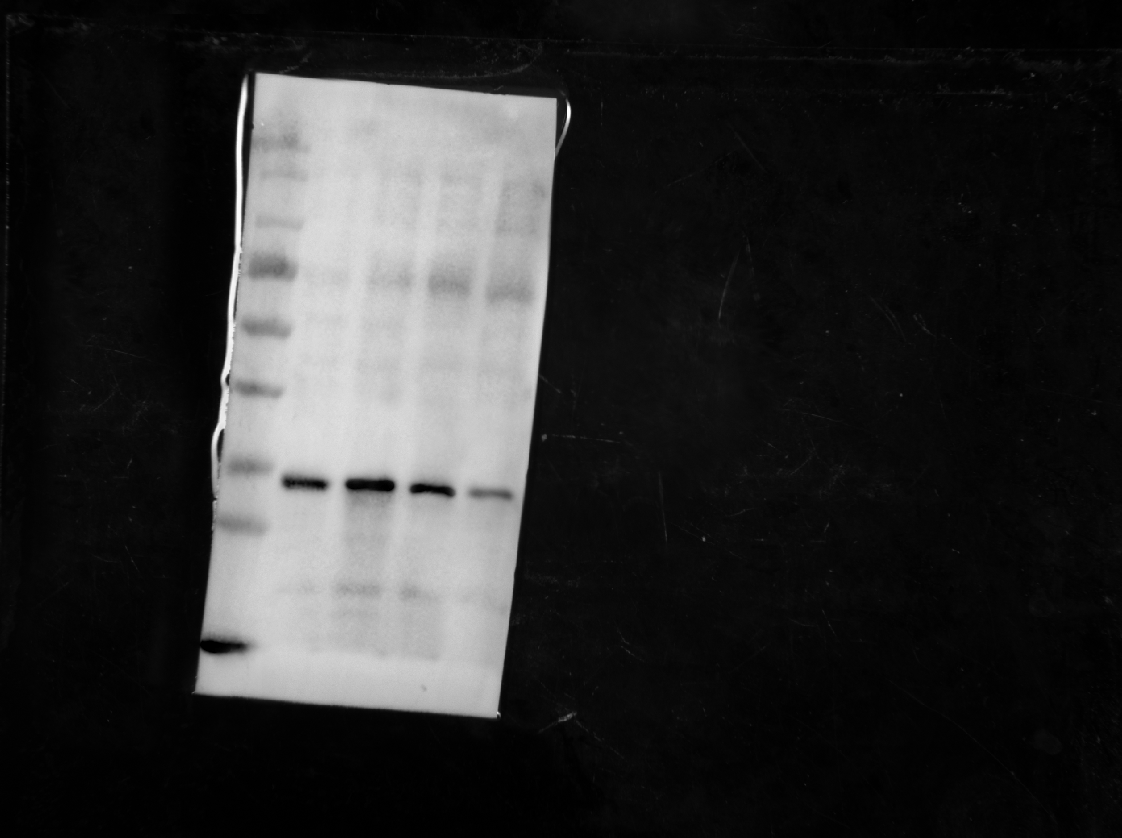
**

**Figure 5A GAPDH**

**
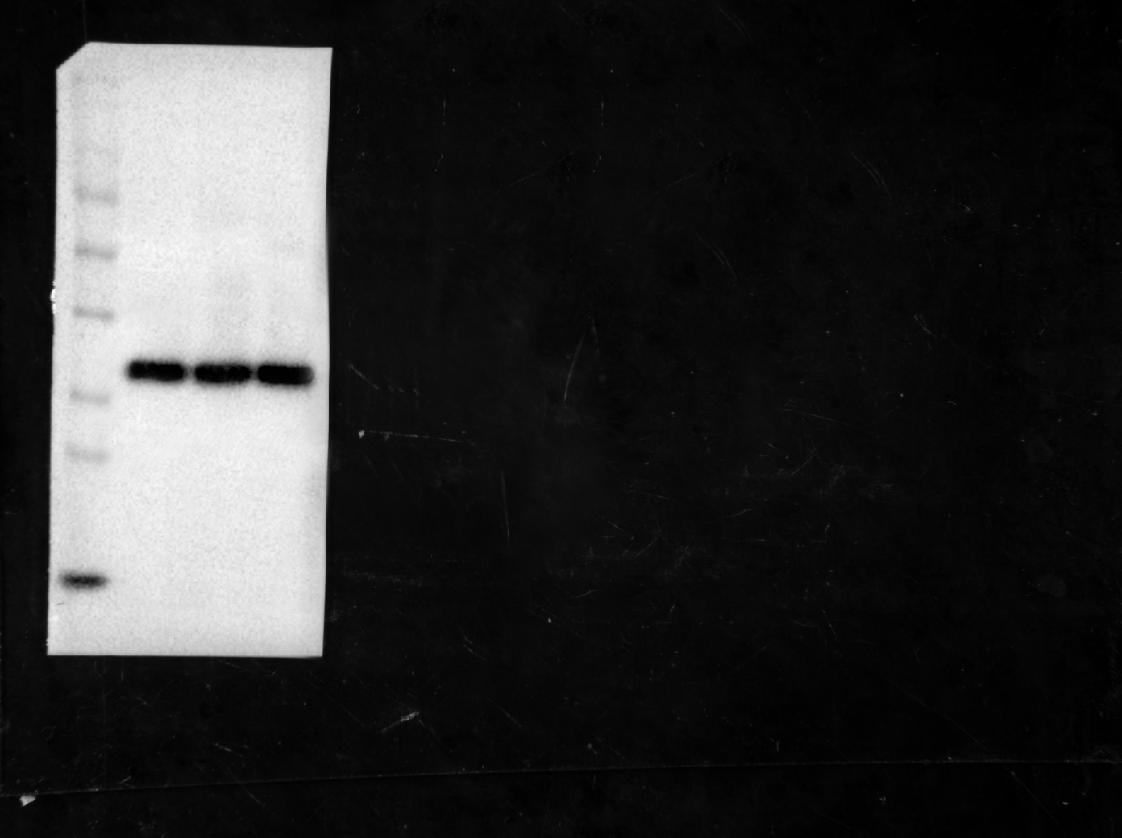
**

**Figure 5A Myd88**

**
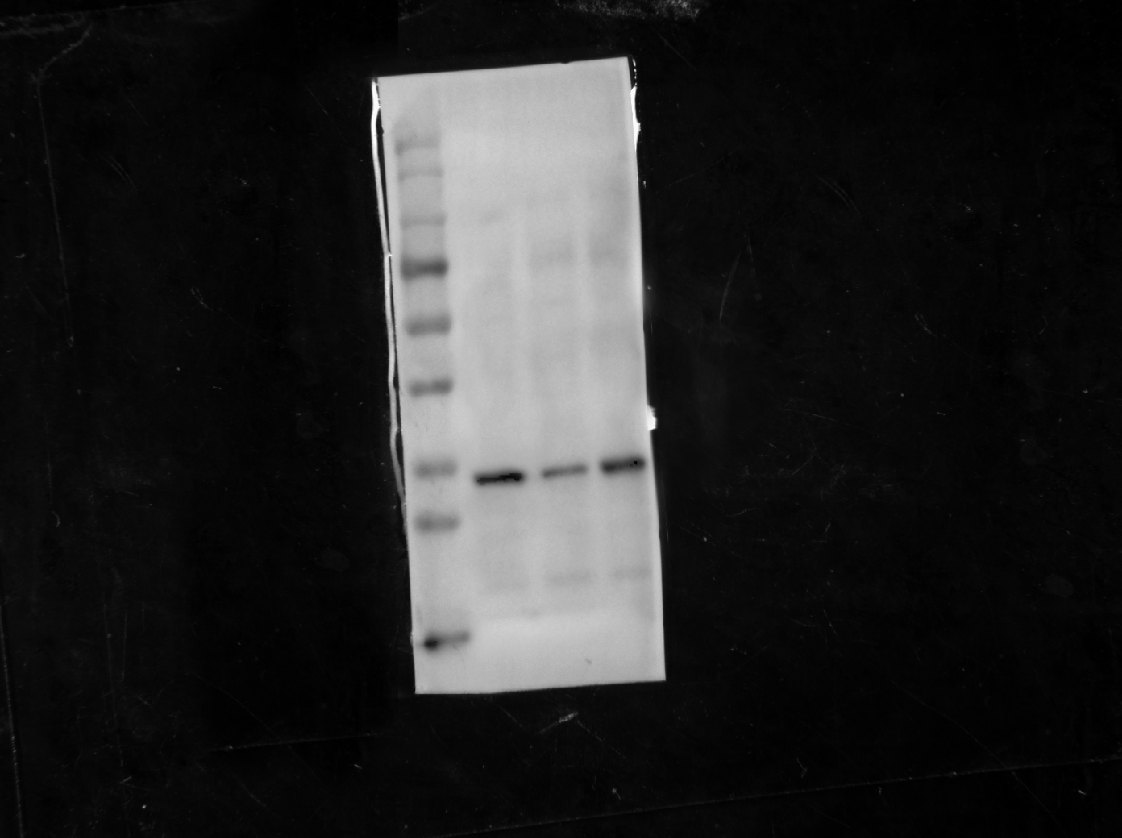
**

**Figure 5A NF-KB**

**
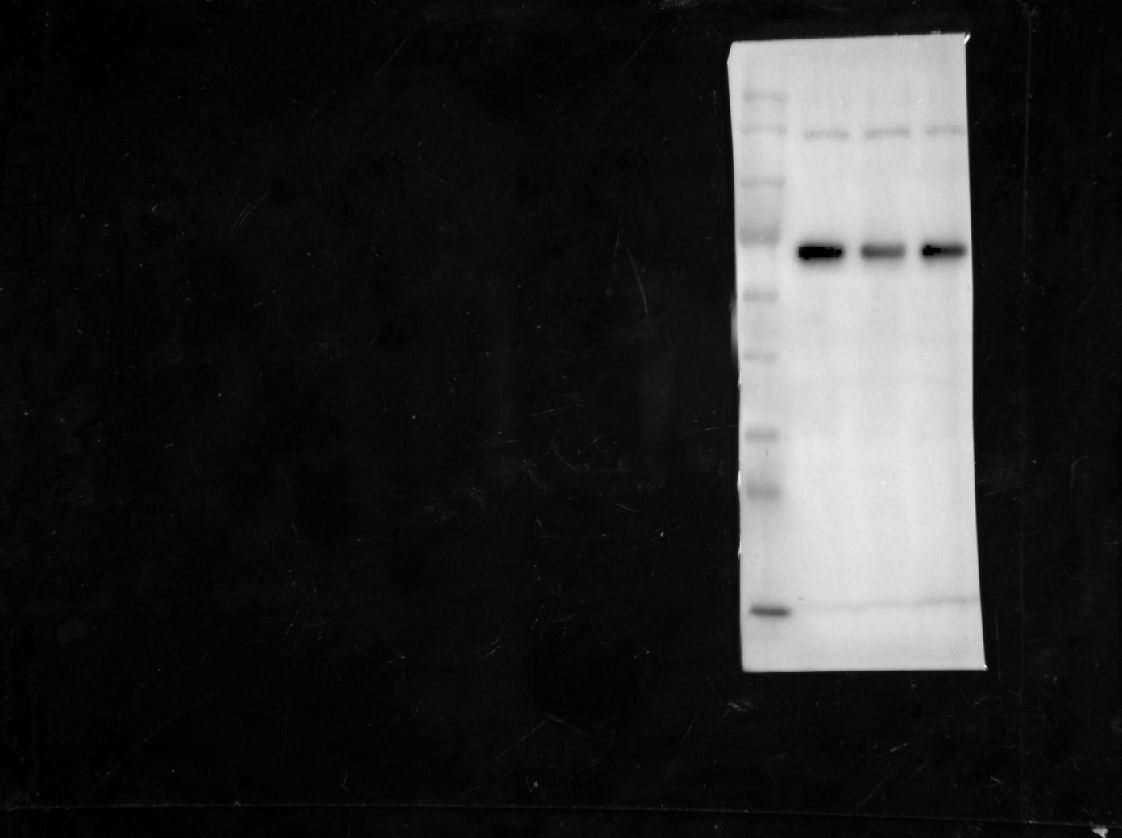
**

**Figure 6E GAPDH**

**
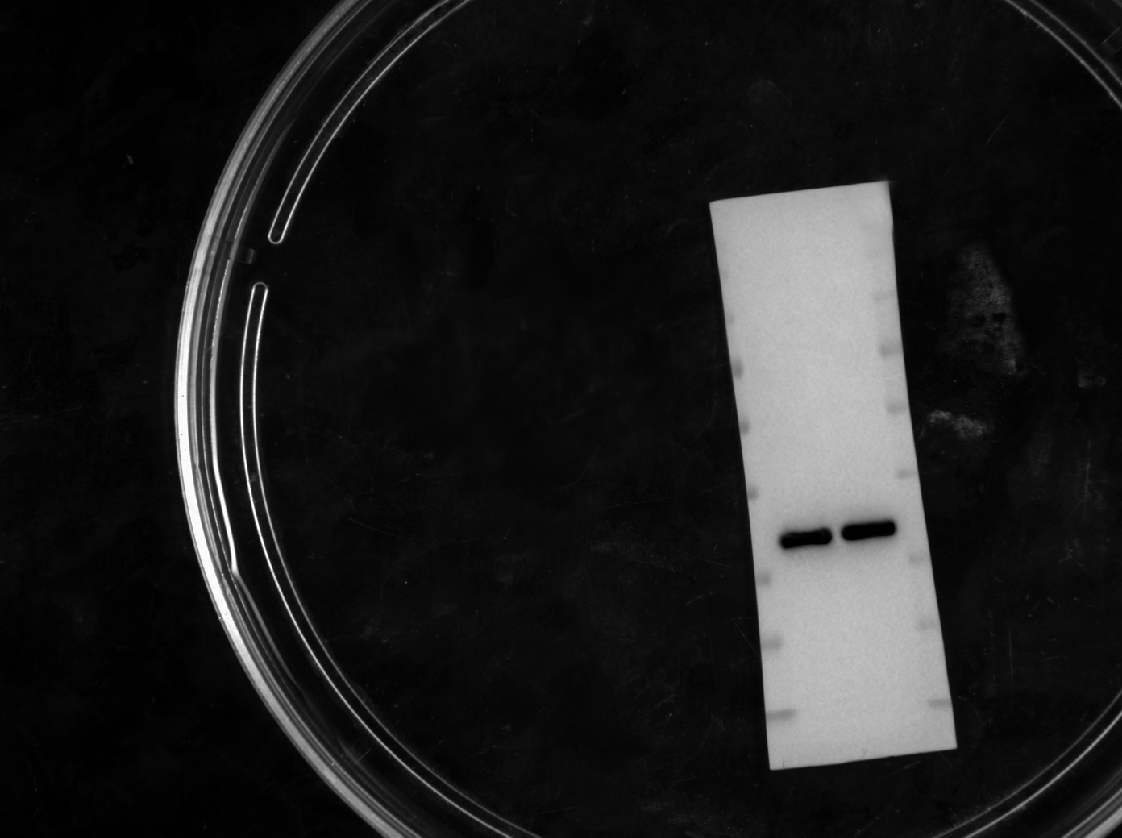
**

**Figure 6E Myd88**

**
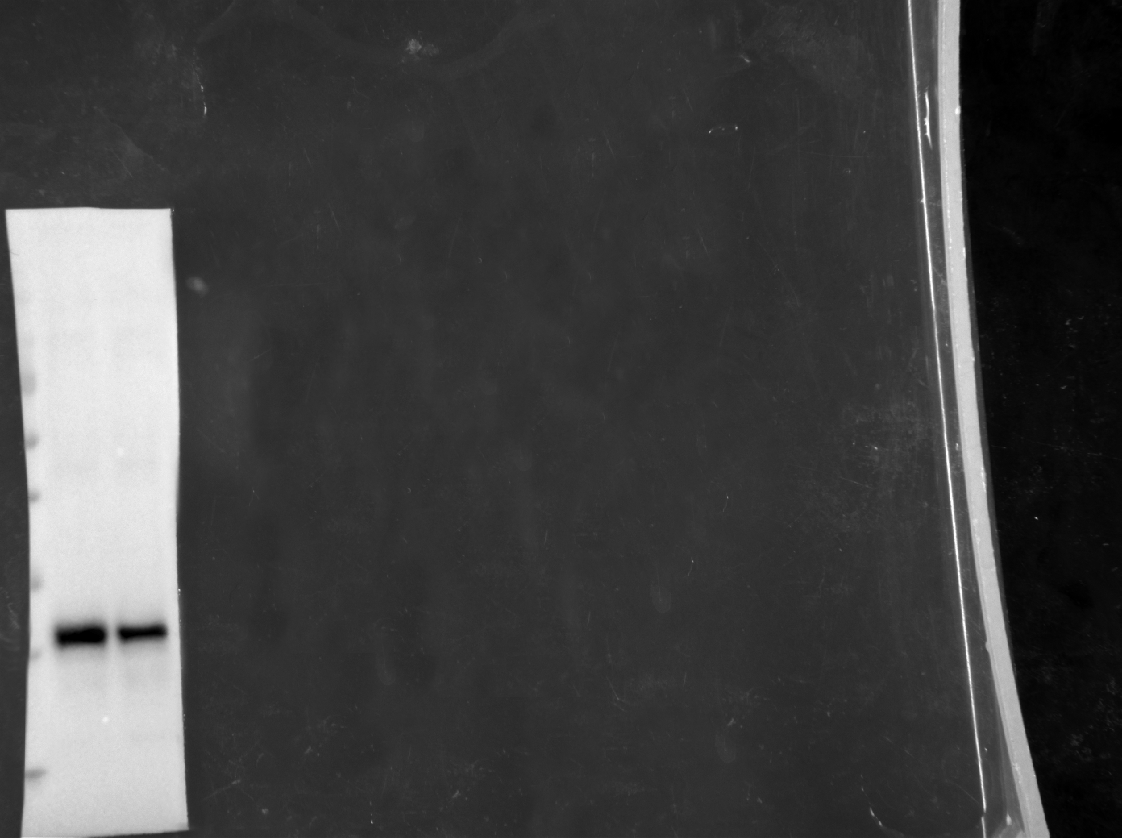
**

**Figure 6E NF-KB**

**
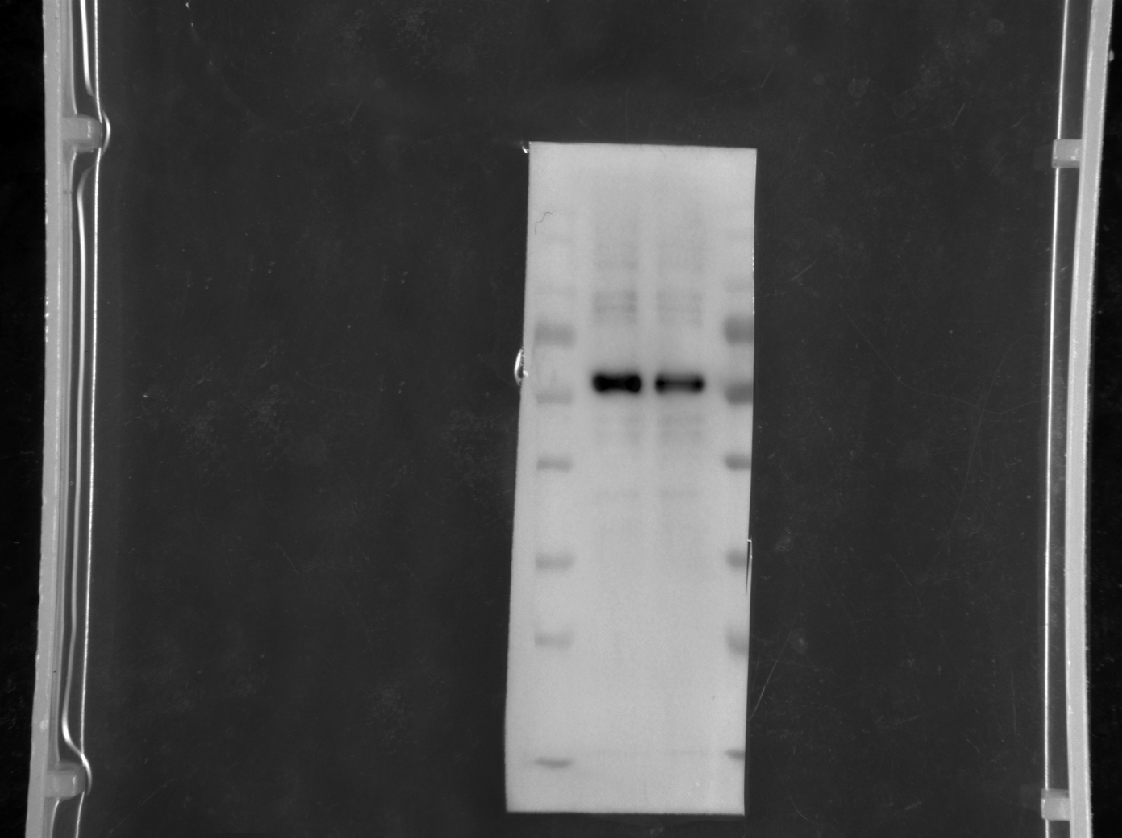
**
